# Low Immunogenicity of Intravesical Phage Therapy for Urogenitary Tract Infections

**DOI:** 10.3390/antibiotics10060627

**Published:** 2021-05-25

**Authors:** Sławomir Letkiewicz, Marzanna Łusiak-Szelachowska, Ryszard Międzybrodzki, Maciej Żaczek, Beata Weber-Dąbrowska, Andrzej Górski

**Affiliations:** 1 Phage Therapy Unit, Hirszfeld Institute of Immunology and Experimental Therapy, Polish Academy of Sciences (HIIET PAS), 53-114 Wrocław, Poland; letkiewicz1@o2.pl (S.L.); beata.weber-dabrowska@hirszfeld.pl (B.W.-D.); andrzej.gorski@hirszfeld.pl (A.G.); 2 Faculty of Health Sciences, Jan Długosz University in Częstochowa, 42-200 Częstochowa, Poland; 3 Bacteriophage Laboratory, Hirszfeld Institute of Immunology and Experimental Therapy, Polish Academy of Sciences (HIIET PAS), 53-114 Wrocław, Poland; marzanna.lusiak-szelachowska@hirszfeld.pl (M.Ł.-S.); maciej.zaczek@hirszfeld.pl (M.Ż.); 4 Department of Clinical Immunology, Transplantation Institute, Medical University of Warsaw, 02-006 Warsaw, Poland; 5 Infant Jesus Hospital, Medical University of Warsaw, 02-005 Warsaw, Poland

**Keywords:** antibiotic resistance, antiphage antibody, immune system, phage therapy, urogenital tract infections

## Abstract

Patients with chronic urinary and urogenital multidrug resistant bacterial infections received phage therapy (PT) using intravesical or intravesical and intravaginal phage administration. A single course of PT did not induce significant serum antibody responses against administered phage. Whilst the second cycle of PT caused a significant increase in antibody levels, they nevertheless remained quite low. These data combined with good therapy results achieved in some patients suggest that this mode of PT may be an efficient means of therapy for urogenital infections and a reliable model for a clinical trial of PT.

## 1. Introduction

Even though no clinical trial carried out so far has confirmed the true value of phage therapy (PT) in combating multidrug resistant bacterial infections, interest in PT has grown and many new reports claiming its efficacy have been published [1]. Among those articles are reports from our group describing promising results achieved in patients with urinary tract infections (UTI), especially in patients with chronic bacterial prostatitis [2,3]. Furthermore, we reported successful use of PT in association with antibiotics in a renal allograft recipient with UTI [4]. A recently published review on PT in UTI summarized currently available data suggesting that this mode of treatment may be an attractive option for those patients [5]. Nevertheless, a randomized, placebo-controlled, double-blind clinical trial of intravesical PT for treating UTI in patients undergoing transurethral resection of the prostate was not found to be superior to placebo bladder irrigation [6]. Therefore, new studies and more data are needed to clarify the value of PT in treating UTI. 

## 2. Materials and Methods

### 2.1. Patients

Patients (11 women and 4 men) with bacterial chronic urinary or urogenital infections underwent phage therapy (PT) between 2016 and 2019 at HIIET Phage Therapy Unit (PTU). Patients used phage preparations selected for treatment on the basis of phage typing (phages indicating a high lytic activity against the patient’s bacterial strain) intravaginally and intravesically or intravesically according to the protocol “Experimental phage therapy of drug-resistant bacterial infections, including MRSA infections” [7]. Phages were administered as follows: intravesically 10 mL two times daily using a urinary catheter Nelaton CH-12; intravaginally and intravesically 10 mL by each route of phage administration two times daily. Eleven patients (7 women and 4 men) underwent one cycle of PT and 4 patients (women) underwent the first cycle of PT with intravaginal and intravesical or intravesical administration of phages (Table 1). Four patients (women) were qualified for the second cycle of PT with intravaginal and intravesical or intravesical administration of phage (Table 2). Patients used phages for 3 days in one cycle of PT or in the first cycle of PT. Then there was a break of 20–61 days before the second cycle of PT, where phages were applied for 3 days. Blood was collected before PT, on the third day of PT and after PT. The blood was centrifuged at 1500× *g* for 10 min and sera were stored at −70 °C. Sera of voluntary blood donors were obtained from the Blood Transfusion Center in Wrocław, Poland. The antiphage activity of sera (AAS) was studied immediately after obtaining sera. Research of AAS in patients undergoing PT at the HIIET PTU and in healthy individuals were performed after obtaining the consent of the Bioethics Committee of the Wrocław Medical University (Poland). All subjects gave written informed consent.

### 2.2. Bacteriophage Preparations

Patients with chronic urinary or urogenital infections used monovalent lysates of bacteriophage preparations of *Escherichia coli*, *Klebsiella pneumoniae*, *Enterococcus faecalis* or *Pseudomonas aeruginosa* based on phage typing (Table 1 and Table 2). Phage lysates had concentrations of 10^6^–10^8^ plaque forming unit/mL (pfu/mL). Healthy donors did not use bacteriophage preparations, but their sera were examined for the presence of antiphage antibodies against bacteriophage preparations *E. coli* phage coli 48/D1, *K. pneumoniae* phage Kl 16/30, *E. faecalis* phage Entc 15/P and *P. aeruginosa* phage Ps F8/PsF8. 

### 2.3. Plate Phage Neutralization Test

The level of AAS of patients undergoing phage therapy and control of healthy individuals was tested with the plate phage neutralization test. The AAS research was performed according to the method described earlier [8]. Undiluted and diluted sera from 1:10 to 1:1500 were tested. A total of 50 µL of the phage with a titer of 1 × 10^6^ pfu/mL were added to each serum dilution (450 µL). Phage titer control was performed by adding 50 µL of the phage with a titer of 1 × 10^6^ pfu/mL to 450 µL of broth. The mixture was incubated for 30 min at 37 °C. After this time, 50 µL of the mixture were taken and added to 4.95 mL of cold broth. Phage titer was examined at the beginning of the phage reaction with the serum and, after the 30 min reaction, by the standard double-agar layer method. A total of 100 µL of the mixture and 200 µL of the bacterial strain were added to 3 mL of 0.7% agar and poured onto agar plates. The plates were incubated for 8 h at 37 °C. AAS was determined as the rate of phage inactivation K (K = 2.3 × (D/T) × log (P0/Pt), where D is the reciprocal of the serum dilution, T is the phage–serum reaction time (30 min), P0 is the phage titer at the beginning of the phage–serum reaction and Pt is the phage titer after time T of the phage–serum reaction). A rate K of less than 5 was considered to be a low level of phage inactivation, a K of between 5 and 18 as a medium level of phage inactivation and above 18 as a high level of phage inactivation.

A statistical analysis for K rate was performed using the Wilcoxon rank sum test (dependent trials). *p* < 0.05 was considered significant.

### 2.4. Categories of the Results of PT

The outcome of PT was assessed according to Międzybrodzki et al. (2012) [7].

Categories A–C were recommended as positive responses to PT:

A—pathogen eradication and/or recovery (eradication confirmed by the results of bacterial cultures; recovery refers to wound healing or complete subsidence of the infection symptoms); B—good clinical result (almost complete subsidence of some infection symptoms, together with a significant improvement of the patient’s general condition after completion of PT); C—clinical improvement (discernible reduction in the intensity of some infection symptoms after completion of PT to a degree not observed before PT, when no treatment was used).

Categories D–G were recommended as inadequate responses to PT:

D—questionable clinical improvement (reduction in the intensity of some infection symptoms to a degree that could also be observed before PT); E—transient clinical improvement (reduction in the intensity of some infection symptoms observed only during application of phage preparations and not after termination of PT); F—no response to treatment (lack of reduction in the intensity of some infection symptoms observed before PT); G—clinical deterioration (exacerbation of symptoms of infection at the end of PT).

## 3. Results

Antiphage activity of sera in 15 patients with chronic urinary or urogenital infections was examined before, during and after PT. The control of AAS was sera from 10 healthy donors showing a low level of AAS against phages: *coli* 48/D1 (mean K rate = 0.01 ± 0.02), Kl 16/30 (mean K rate = 0.01 ± 0.02), Entc 15/P (mean K rate = 0.07 ± 0.08) and Ps F8/PsF8 (mean K rate = 0.11 ± 0.19).

Fifteen patients using intravaginal and intravesical or intravesical phages received one cycle of 3 days of PT (7 women and 4 men) or the first cycle of 3 days of PT (4 women) (Table 1). Before phage therapy, these patients had low AAS levels (mean K rate = 0.13 ± 0.43). A low level of antibodies in this group of patients was demonstrated on the third day of phage therapy (mean K rate = 0.15 ± 0.51). In this group of patients, the increase in the K rate during PT compared to the K rate before PT was statistically insignificant (Wilcoxon test; *p* = 0.10). In the period of 14–61 days after the therapy, the level of antibodies was still low (mean K rate = 0.12 ± 0.42). Four women from the first group, who underwent the first cycle of PT, qualified for the second cycle of PT after a 20–61 day break. Four women who had two cycles of PT, before the second cycle of therapy, had low AAS levels (mean K rate = 0.36 ± 0.78). (Table 2). In the second cycle of intravaginal and intravesical or intravesical therapy, the level of AAS on the third day of phage administration was still low (mean K rate = 0.49 ± 0.97). Nevertheless, the increase in the K rate during PT compared to the K rate before PT was statistically significant (Wilcoxon test; *p* = 0.04). 

Analysis of clinical outcome of PT was performed in 11 women with intravaginal and intravesical or intravesical application of phages and in 4 men with intravesical application of phages. Eleven women underwent one cycle of PT or the first cycle of PT intravaginally and intravesically or only intravesically and six of them (54.5%) had A–C results of PT. Four men underwent one cycle of PT intravesically and all men had F results of PT. The results of bacteriological assays prior to, during and after PT are depicted in Table 1 and Table 2. No significant side effects of PT were noted.

## 4. Discussion

Our recent review presents the current status and perspectives of PT [1]. In the past decade, interest in PT has rapidly grown and reports on its successful use in treating multidrug-resistant bacterial infections are published almost every month [9,10,11,12,13,14,15,16,17]. Likewise, several reviews on PT are published each year. However, there is a growing gap between the expansion of PT carried out as compassionate use (experimental therapy) and the lack of reliable data derived from clinical trials of PT performed according to the required standards of Evidence-Based Medicine and EMA (FDA). Notably, no double-blind randomized clinical trial has provided conclusive data confirming the real therapeutic value of the therapy. Therefore, there is an urgent need for a well-planned clinical trial that could determine whether indeed PT can offer a reliable weapon against antibiotic-resistant bacterial infections. 

The route of phage administration and neutralizing antibody production against phages constitute major factors which may determine the success of such a trial. Our present report extends our earlier data indicating that the production of anti-phage antibodies depends on the route of phage administration [18]. In fact, intravesical phage administration induced only minimal humoral responses. Those responses increased significantly during repeat phage administration although their level remained low, especially when compared to levels reached in response to topical (e.g., intrawound and intrafistular administration [8,18]. Furthermore, only weak anti-phage antibody production combined with a lack of significant side effects and good results of the therapy in some patients with UTI appears to be promising.

In conclusion, we report safety and low immunogenicity of intravesicular PT, which suggests that further clinical trials using this approach are warranted.

## Figures and Tables

**Table 1:** Patients with chronic urinary or urogenital infections undergoing one cycle or the first cycle of phage therapy.

Patient No. ^a^	Type of Infection	Route of Phage Administration	Target Pathogen	Phage Used in PT	Phage Inactivation (K) ^b^				Clinical Outcome of PT ^c^	Bacterial Load of the Target Pathogen in Urine (CFU/mL)			Comment
					before PT	on the 3rd day of PT	after PT	Days after PT		before PT ^d^	on the 3rd day of PT	after PT	
1 (f)	Chronic urinary tract infection	Intravesical and intravaginal	*E. coli*	coli A11/58c	0.15	0.05	0.02	21	A	10^6^	10^5^	neg	*C. koseri* was detected in urine at 10^6^ CFU/mL after PT.
2 (f)	Chronic urinary tract and vaginal infection	Intravesical and intravaginal	*E. coli*	coli A11/58c	0.00	0.009	0.007	17	A ^1^	10^6^	10^6^	10^5^	Additionally, *E. faecalis* was detected in urine at 10^5^ CFU/mL after PT. *E. coli* was isolated (heavy growth) from the vagina both before and after PT.
3 (f)	Chronic urogenital infection	Intravesical and intravaginal	*K. pneumoniae*	Kl 53N/1920	0.007	0.007	0.007	30	C ^2^	10^6^	10^5^	10^6^	*K. pneumoniae* at 10^6^ CFU/mL was also detected in urine culture 12 days after PT. It was isolated (moderate growth) from the vagina before and 12 days after PT.
4 (f)	Chronic urinary tract and vaginal infection	Intravesical and intravaginal	*K. pneumoniae* *E. faecalis*	Kl 53N/1920 EF1/1679Ł	2.01 0.09	2.39 0.14	1.95 0.12	20	C	10^3^ neg	not tested	10^2^ neg	Both *K. pneumoniae* and *E. faecalis* were isolated (heavy growth) from the vagina 40 days before PT cycle. *E. coli* was detected in urine at 10^2^–10^3^ CFU/mL on a day when PT was started as swell during and after PT.
5 (f)	Chronic urogenital infection	Intravesical and intravaginal	*E. coli*	coli 99/13127	0.007	0.05	0.006	20	E	10^5^	10^5^	10^5^	*E. coli* was isolated (heavy growth) from the vagina both before and after PT.
6 (f)	Chronic urogenital infection	Intravesical and intravaginal	*E. coli*	coli 98/13127	0.14	0.14	0.06	20	C	10^6^	10^3^	10^5^	*S. agalactiae* was isolated from urine (10^5^–10^6^ CFU/mL) during and after PT.
7 (f)	Chronic urogenital infection	Intravesical and intravaginal	*E. coli*	coli A11/58c	0.13	0.09	0.01	61	F	10^4^	10^6^	10^4^	*E. coli* was isolated (very heavy growth) from the vagina both before, during and after PT.
8 (f)	Chronic urinary tract infection	Intravesical	*K. pneumoniae*	Kl 53N/1920	0.002	0.002	0.003	14	D	10^5^	10^6^	10^5^	Additionally, *E. coli* was detected in urine at 10^5^ CFU/mL after PT.
9 (f)	Chronic urinary tract infection	Intravesical	*E. coli* *K. pneumoniae*	coli 54/181 Kl 53N/1920	0.007 0.008	0.008 0.01	0.007 0.009	26	F	10^5^	10^5^	10^4^	*K. pneumoniae* at 10^5^ CFU/mL was also detected in urine culture 11 days after PT. Additionally, *E. coli* was detected in urine at 10^4^ CFU/mL after PT.
10 (f)	Chronic urinary tract infection	Intravesical	*P. aeruginosa* *E. coli*	Ps1N/734 coli 77/850	0.01 0.02	0.01 0.04	0.00 0.00	27	F	10^5^ neg	10^5^ neg	10^6^ neg	*P. aeruginosa* as well as *E. coli* were detected at 10^6^ CFU/mL in urine culture 85 days before PT.
11 (f)	Chronic urinary tract and vaginal infection	Intravesical	*K. pneumoniae*	Kl 24/24	0.00	0.00	0.06	60	A ^3^	10^6^	10^5^	neg	
12 (m)	Chronic urinary tract infection	Intravesical	*K. pneumoniae* *K. pneumoniae*	Kl 16/30 K22.KC/28483	0.05 0.00	0.11 0.002	0.02 0.10	21	F	10^6^	10^6^	10^6^	*P. mirabilis* was transiently detected at 10^6^ CFU/mL in urine before and after PT.
13 (m)	Chronic urinary tract infection	Intravesical	*K. pneumoniae* *K. pneumoniae*	Kl 28N/1115 Kl 40N/679	0.04 0.002	0.05 0.003	0.03 0.02	42	F	10^6^	10^6^	10^5^	
14 (m)	Chronic urinary tract infection	Intravesical	*E. coli* *K. variicola*	coli 98/13127 Kl 52N/1920	0.00 0.02	0.03 0.03	0.01 0.03	27	F ^4^	10^6^ neg	10^6^ neg	neg 10^6^	*E. coli* and *K. variicola* were detected in urine 48 days before PT.
15 (m)	Chronic urinary tract infection	Intravesical	*E. coli*	coli 126/2031	0.02	0.05	0.01	26	F	10^6^	10^5^	10^5^	
			K range: Mean K ± SD: Wilcoxon test:		0.00–2.01 0.13 ± 0.43	0.00–2.39 0.15 ± 0.51 ^5^ *p* = 0.10	0.00–1.95 0.12 ± 0.42 ^6^ *p* = 0.27						

Abbreviations: *f*, female; *m*, male; *PT*, phage therapy; *SD*, standard deviation; *neg*, bacterial titer below detection limit (10^3^ CFU/mL). ^a^ Patients No. 3, 4, 7, and 10 underwent the first cycle out of two cycles of PT. The remaining patients underwent one cycle of PT. ^b^ Rate of phage inactivation: K < 5, low neutralization of phages; K = 5–18, medium neutralization of phages; K > 18, high neutralization of phages. ^c^ Clinical outcome: A–C, positive responses to PT; D–G, inadequate responses to PT. ^d^ Urine sample was usually collected via intravesical catheter just before starting the phage application. ^1^ Pathogen eradication from vagina only. ^2^ Significant change in antibiotic and phage sensitivity of bacterial strain isolated after treatment. ^3^ Pathogen eradication from urinary tract only. ^4^ *E. coli* was transiently eradicated after treatment. ^5^ Statistically insignificant increase in the K rate during PT compared to the K rate before PT. ^6^ Statistically insignificant decrease in the K rate after PT compared to the K rate before PT.

**Table 2:** Patients with chronic urinary or urogenital infections undergoing two cycles of phage therapy.

Patient No. ^a^	Type of Infection	Route of Phage Administration	Target Pathogen	Phage Used in PT	Phage Inactivation (K) ^b^				Clinical Outcome of PT Cycle ^c^	Bacterial Load of the Target Pathogen in Urine (CFU/mL)			Comment
					before PT Cycle	on the 3rd day of PT Cycle	after PT Cycle	Days after PT Cycle		before PT Cycle ^d^	on the 3rd day of PT Cycle	after PT Cycle	
3 (f)	Chronic urogenital infection	The first PT cycle: intravesical and intravaginal	*K. pneumoniae*	Kl 53N/1920	0.007	0.007	0.007	30	C ^1^	10^6^	10^5^	10^6^	*K. pneumoniae* at 10^6^ CFU/mL was also detected in urine culture 12 days after the first PT cycle. It was isolated (moderate growth) from the vagina before and 12 days after the first PT cycle.
		The second PT cycle (started 30 days after the first one): intravesical and intravaginal	*K. pneumoniae*	Kl 53N/1920	0.007	0.04	0.007	28	not possible to evaluate	10^6^	10^6^	not tested	
4 (f)	Chronic urinary tract and vaginal infection	The first PT cycle: intravesical and intravaginal	*K. pneumoniae* *E. faecalis*	Kl 53N/1920 EF1/1679Ł	2.01 0.09	2.39 0.14	1.95 0.12	20	C	10^3^ neg	not tested	10^2^ neg	Both *K. pneumoniae* and *E. faecalis* were isolated (heavy growth) from the vagina 40 days before the first PT cycle. *E. coli* was detected in urine at 10^2^–10^3^ CFU/mL on a day when PT was started as swell during and after the first PT cycle.
		The second PT cycle (started 20 days after the first one): intravesical and intravaginal	*K. pneumoniae* *E. coli* *E. faecalis*	Kl 53N/1920 coli A11/58c EF1/1679Ł	1.95 0.07 0.12	2.47 0.12 0.25	1.75 0.09 0.23	12	A ^2^	10^2^ 10^2^ neg	10^2^ 10^2^ neg	10^4^ neg neg	Both *K. pneumoniae* and *E. coli* were isolated (heavy growth) from the vagina on a day when the second PT cycle was started. Only physiological flora was detected in a vaginal swab after PT.
7 (f)	Chronic urogenital infection	The first PT cycle: intravesical and intravaginal	*E. coli*	coli A11/58c	0.13	0.09	0.01	61	F	10^4^	10^6^	10^4^	*E. coli* was isolated (very heavy growth) from the vagina both before, during and after the first PT cycle.
		The second PT cycle (started 61 days after the first one): intravesical and intravaginal	*E. coli*	coli A11/58c	0.01	0.05	0.03	12	F	10^4^	10^6^	10^3^	*E. coli* was isolated (heavy growth) from the vagina both before, during and after the second PT cycle.
10 (f)	Chronic urinary tract infection	The first PT cycle: intravesical	*E. coli* *P. aeruginosa*	coli 77/850 Ps1N/734	0.02 0.01	0.04 0.01	0.00 0.00	27	F	10^5^ neg	10^5^ neg	10^6^ neg	*P. aeruginosa* as well as *E. coli* were detected at 10^6^ CFU/mL in urine culture 85 days before PT.
		The second PT cycle (started 27 days after the first one): intravesical	*E. coli*	coli 126/2031	0.00	0.00	not tested		A	10^6^	not tested	neg	Concomitant antibiotic treatment with ciprofloxacin was applied during this PT cycle.
	The first PT cycle		K range: Mean K ± SD: Wilcoxon test:		0.007–2.01 0.38 ± 0.80	0.007–2.39 0.44 ± 0.95 ^3^ *p* = 0.27	0.00–1.95 0.35 ± 0.78 ^4^ *p* = 0.22						
	The second PT cycle		K range: Mean K ± SD: Wilcoxon test:		0.00–1.95 0.36 ± 0.78	0.00–2.47 0.49 ± 0.97 ^5^ *p* = 0.04							
			K range: Mean K ± SD: Wilcoxon test:		0.007–1.95 0.43 ± 0.85		0.007–1.75 0.42 ± 0.74 ^6^ *p* = 0.71						

Abbreviations: *f*, female; *PT*, phage therapy; *SD,* standard deviation; *neg*, bacterial titer below detection limit (<10^3^ CFU/mL). ^a^ Patients No. 3, 4, 7, and 10 underwent the first cycle out of two cycles of PT. The remaining patients underwent one cycle of PT. ^b^ Rate of phage inactivation: K < 5, low neutralization of phages; K = 5–18, medium neutralization of phages; K > 18, high neutralization of phages. ^c^ Clinical outcome: A–C, positive responses to PT; D–G, inadequate responses to PT. ^d^ Urine sample was usually collected via intravesical catheter just before starting the phage application. ^1^ Significant change in antibiotic and phage sensitivity of bacterial strain isolated after treatment. ^2^ Pathogen eradication from vagina only. ^3^ Statistically insignificant increase in the K rate during the first PT cycle compared to the K rate before starting PT. ^4^ Statistically insignificant decrease in the K rate after the first PT cycle compared to the K before PT. ^5^ Statistically significant increase in the K rate during the second PT cycle compared to the K rate before the second PT cycle. ^6^ Statistically insignificant decrease in the K rate after the second PT cycle compared to the K before PT (for patients 3, 4 and 7).

## Data Availability

The data presented in this study are derived from personal patients’ medical records maintained at the Phage Therapy Unit of the Medical Centre as well as Bacteriophage Laboratory of the Institute of Immunology and Experimental Therapy Polish Academy of Sciences in Wrocław, Poland. Those data are not publicly available due to privacy and legal issues (The General Data Protection Regulation (EU) 2016/679 and Act on the rights of the patient and the Patient’s Rights Ombudsman from 6 November 2008).
